# Supplementary material for: Reaching silicon-based NEMS performances with 3D printed nanomechanical resonators
Source: Nat Commun. 2021 Oct 19;12:6080. doi: 10.1038/s41467-021-26353-1 (PMC8526607; doi:10.1038/s41467-021-26353-1)
Supplement: Supplementary file 1 — Supplementary Information [file 41467_2021_26353_MOESM1_ESM.pdf]

SUPPLEMENTARY INFORMATION

Reaching silicon-based NEMS performances with 3D printed nanomechanical resonators

Stefano Stassi[1*], Ido Cooperstein[2], Mauro Tortello[1], Candido Fabrizio Pirri[1], Shlomo Magdassi[2*], Carlo Ricciardi[1]

[1]Department of Applied Science and Technology, Politecnico di Torino, Corso Duca Degli Abruzzi, 24, 10129 Torino, Italy

[2]Institute of Chemistry, The Hebrew University of Jerusalem, Jerusalem, Israel 91904

*Corresponding Authors: stefano.stassi@polito.it, magdassi@mail.huji.ac.il

Supplementary Note 1 – Quality factor review

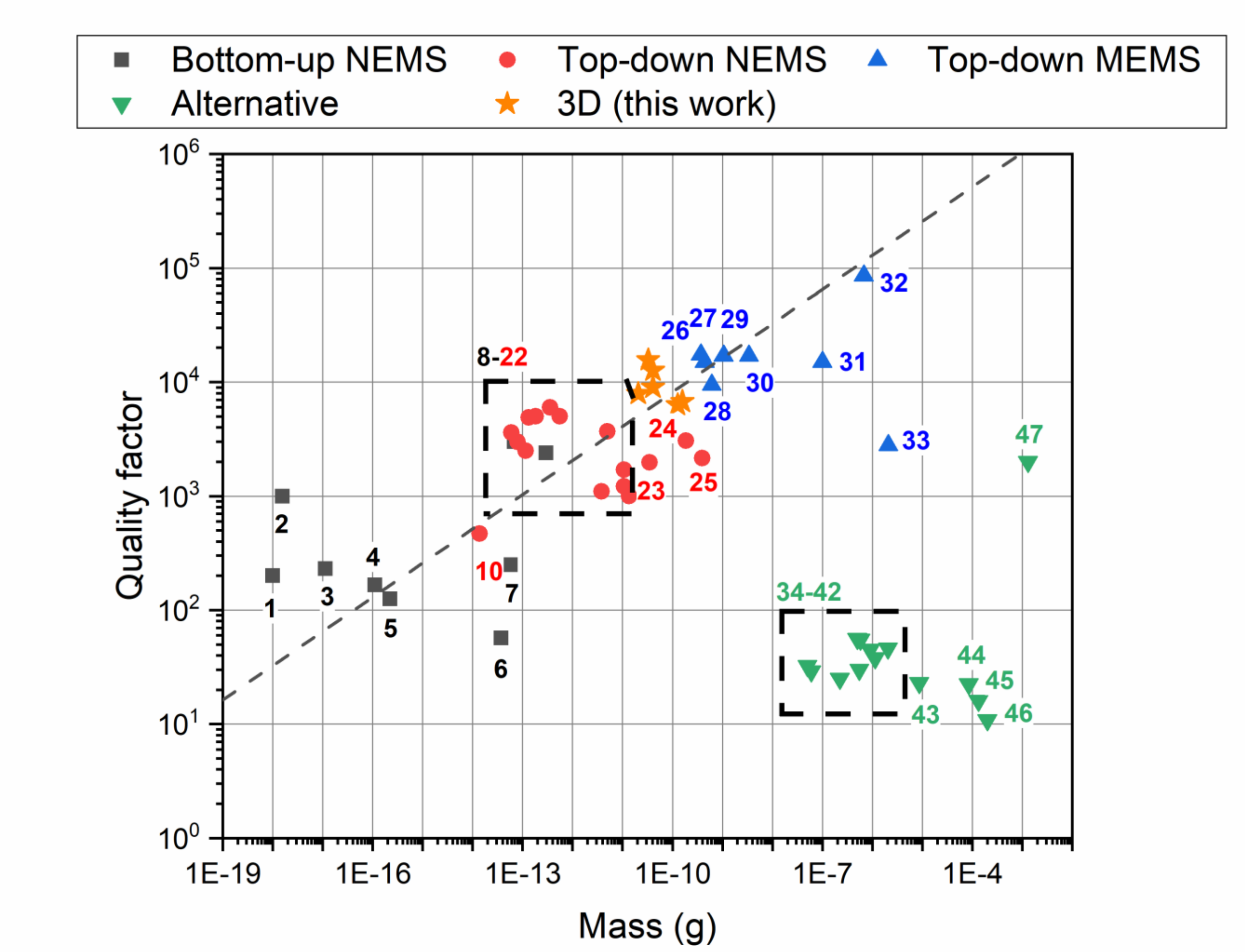

Supplementary Figure S1. Plot of Figure 1 of the manuscript with the mapping of the reference work from which quality factor data are extracted. The reference articles are reported below. The dashed rectangles represent the zoomed area of Supplementary Figure S2 and S3.

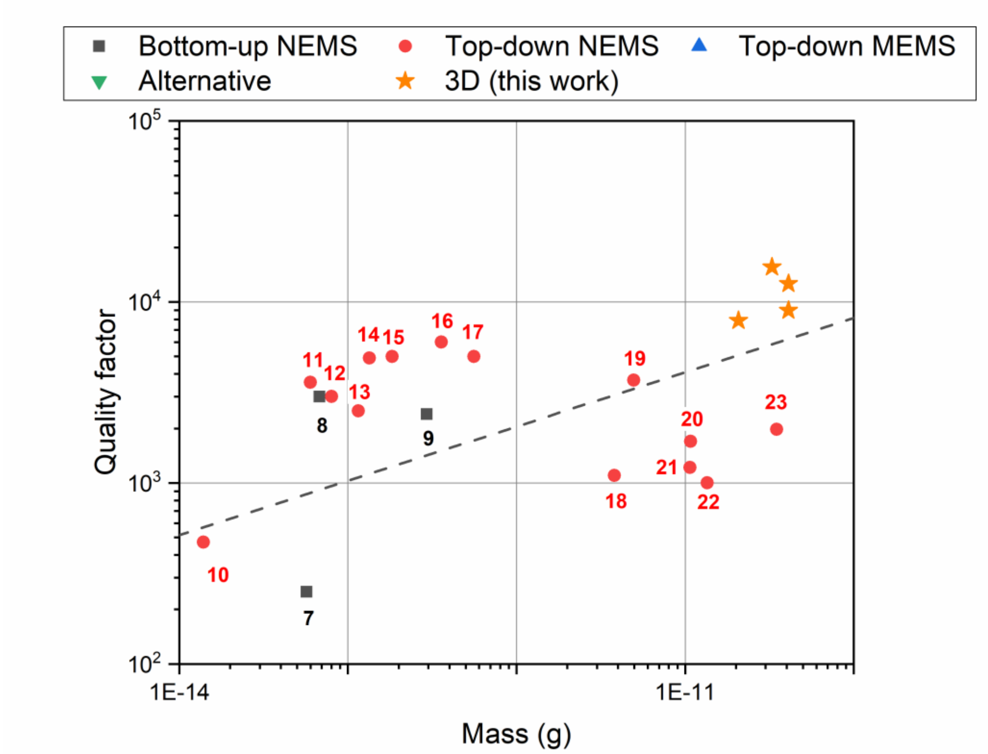

Supplementary Figure S2. Zoom of Supplementary Figure S1 containing reference points from 8 to 22.

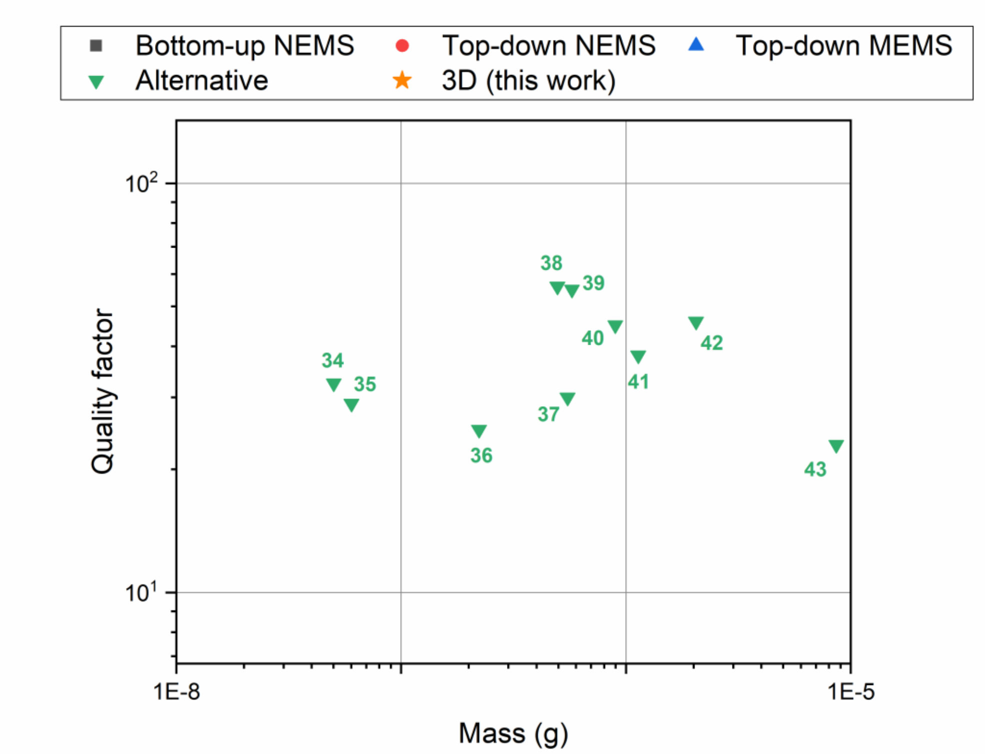

Supplementary Figure S3. Zoom of Supplementary Figure S1 containing reference points from 34 to 43.

Figure 1 of the manuscript reports the quality factor values of several mechanical resonators extrapolated from literature work and compare to the performance of our devices. Only quality factor measured at room temperature are reported. The devices are divided in 4 different categories related to fabrication technology and resonator dimensions. Three categories are somehow related to standard lithographic technology: bottom-up NEMS, top-down NEMS, and MEMS resonators, while the fourth is represented by devices fabricated with alternative techniques such as like screen printing, hot embossing, microinjection molding, solvent casting, nanoimprinting, microfluidic approach, and 3D printing. Below is reported the reference list of the numbered points of Supplementary Figure S1, S2 and S3 (some works presented more than one type of resonator).

BOTTOM-UP NEMS

1. Chiu, H. Y., Hung, P., Postma, H. W. C. & Bockrath, M. Atomic-scale mass sensing using carbon nanotube resonators. Nano Lett. 8, 4342-4346 (2008).
2. Jensen, K., Kim, K. & Zettl, A. An atomic-resolution nanomechanical mass sensor. Nature Nanotechnology 3, 533-537 (2008).
3. Eichler, A., Del Álamo Ruiz, M., Plaza, J. A. & Bachtold, A. Strong coupling between mechanical modes in a nanotube resonator. Phys. Rev. Lett. 109 (2012).
4. Morell, N. et al. High Quality Factor Mechanical Resonators Based on WSe_2 Monolayers. Nano Lett. 16, 5102-5108 (2016).
5. Chen, C. et al. Performance of monolayer graphene nanomechanical resonators with electrical readout. Nature Nanotechnology 4, 861-867 (2009).
6. Robinson, J. T. et al. Wafer-scale reduced graphene oxide films for nanomechanical devices. Nano Lett. 8, 3441-3445 (2008).
7. Barton, R. A. et al. High, size-dependent quality factor in an array of graphene mechanical resonators. Nano Lett. 11, 1232-1236 (2011).
8. De Alba, R. et al. Tunable phonon-cavity coupling in graphene membranes. Nature Nanotechnology 11, 741-746 (2016).
9. Stassi, S. et al. Nanomechanical DNA resonators for sensing and structural analysis of DNA-ligand complexes. Nat. Commun. 10, 1690 (2019).

TOP-DOWN NEMS

10. Bartsch, S. T., Rusu, A. & Ionescu, A. M. Phase-locked loop based on nanoelectromechanical resonant-body field effect transistor. Appl. Phys. Lett. 101 (2012).
11. Sansa, M., Fernández-Regúlez, M., Llobet, J., San Paulo, Á. & Pérez-Murano, F. High-sensitivity linear piezoresistive transduction for nanomechanical beam resonators. Nat. Commun. 5 (2014). *Bridge bottom-up.*
12. Ramos, D. et al. Optomechanics with silicon nanowires by harnessing confined electromagnetic modes. Nano Lett. 12, 932-937 (2012).
13. Feng, X. L., White, C. J., Hajimiri, A. & Roukes, M. L. A self-sustaining ultrahigh-frequency nanoelectromechanical oscillator. Nature Nanotechnology 3, 342-346 (2008).
14. Sansa, M., Fernández-Regúlez, M., Llobet, J., San Paulo, Á. & Pérez-Murano, F. High-sensitivity linear piezoresistive transduction for nanomechanical beam resonators. Nat. Commun. 5 (2014). *Bridge top-down.*
15. Yang, Y. T., Callegari, C., Feng, X. L., Ekinci, K. L. & Roukes, M. L. Zeptogram-scale nanomechanical mass sensing. Nano Lett. 6, 583-586 (2006).
16. Sansa, M. et al. Frequency fluctuations in silicon nanoresonators. Nature Nanotechnology 11, 552-558 (2016).
17. Mile, E. et al. In-plane nanoelectromechanical resonators based on silicon nanowire piezoresistive detection. Nanotechnology 21 (2010).
18. Gray, J. M., Bertness, K. A., Sanford, N. A. & Rogers, C. T. Low-frequency noise in gallium nitride nanowire mechanical resonators. Appl. Phys. Lett. 101 (2012).
19. Cha, J. & Daraio, C. Electrical tuning of elastic wave propagation in nanomechanical lattices at MHz frequencies. Nature Nanotechnology 13, 1016-1020 (2018). *Device with electrodes.*
20. Cha, J. & Daraio, C. Electrical tuning of elastic wave propagation in nanomechanical lattices at MHz frequencies. Nature Nanotechnology 13, 1016-1020 (2018). *Device without electrodes.*
21. Villanueva, L. G. et al. A nanoscale parametric feedback oscillator. Nano Lett. 11, 5054-5059 (2011).
22. Verd, J. et al. Monolithic CMOS MEMS oscillator circuit for sensing in the attogram range. IEEE Electron Device Letters 29, 146-148 (2008).
23. Gagino, M. et al. Suspended Nanochannel Resonator Arrays with Piezoresistive Sensors for High-Throughput Weighing of Nanoparticles in Solution. ACS Sensors 5, 1230-1238 (2020).
24. Villanueva, L. G. et al. Nonlinearity in nanomechanical cantilevers. Physical Review B - Condensed Matter and Materials Physics 87 (2013). *Cantilever aspect-ratio AR 5.*
25. Villanueva, L. G. et al. Nonlinearity in nanomechanical cantilevers. Physical Review B - Condensed Matter and Materials Physics 87 (2013). *Cantilever aspect-ratio AR 13.*

TOP-DOWN MEMS

26. Olcum, S. et al. Weighing nanoparticles in solution at the attogram scale. Proceedings of the National Academy of Sciences of the United States of America 111, 1310-1315 (2014). *Cantilever 22.5 μm long.*
27. Olcum, S. et al. Weighing nanoparticles in solution at the attogram scale. Proceedings of the National Academy of Sciences of the United States of America 111, 1310-1315 (2014). *Cantilever 27 μm long.*
28. Olcum, S. et al. Weighing nanoparticles in solution at the attogram scale. Proceedings of the National Academy of Sciences of the United States of America 111, 1310-1315 (2014). *Cantilever 37.5 μm long.*
29. Olcum, S. et al. Weighing nanoparticles in solution at the attogram scale. Proceedings of the National Academy of Sciences of the United States of America 111, 1310-1315 (2014). *Cantilever 50 μm long.*
30. Ramos, D., Malvar, O., Davis, Z. J., Tamayo, J. & Calleja, M. Nanomechanical Plasmon Spectroscopy of Single Gold Nanoparticles. Nano Lett. 18, 7165-7170 (2018).
31. Burg, T. P. et al. Weighing of biomolecules, single cells and single nanoparticles in fluid. Nature 446, 1066-1069 (2007).
32. Stassi, S. et al. Large-scale parallelization of nanomechanical mass spectrometry with weakly-coupled resonators. Nat. Commun. 10 (2019).
33. Calmo, R. et al. Monolithic glass suspended microchannel resonators for enhanced mass sensing of liquids. Sensors and Actuators, B: Chemical, 298-303 (2019).

ALTERNATIVE APPROACHES

34. McFarland, A. W., Poggi, M. A., Bottomley, L. A. & Colton, J. S. Production and characterization of polymer microcantilevers. Rev. Sci. Instrum. 75, 2756-2758 (2004).
35. Alsharif, N. et al. Design and Realization of 3D Printed AFM Probes. Small 14 (2018).
36. Yoon, Y., Chae, I., Thundat, T. & Lee, J. Hydrogel Microelectromechanical System (MEMS) Resonators: Beyond Cost-Effective Sensing Platform. Advanced Materials Technologies 4 (2019). *Cantilever.*
37. Yoon, Y., Chae, I., Thundat, T. & Lee, J. Hydrogel Microelectromechanical System (MEMS) Resonators: Beyond Cost-Effective Sensing Platform. Advanced Materials Technologies 4 (2019). *Bridge.*
38. Keller, S. S. et al. Fabrication of biopolymer cantilevers using nanoimprint lithography. Microelectron. Eng. 88, 2294-2296 (2011).
39. Shiraishi, N., Ikehara, T., Dao, D. V., Sugiyama, S. & Ando, Y. Fabrication and testing of polymer cantilevers for VOC sensors. Sensors and Actuators, A: Physical 202, 233-239 (2013). *PMMA Cantilever.*
40. Shiraishi, N., Ikehara, T., Dao, D. V., Sugiyama, S. & Ando, Y. Fabrication and testing of polymer cantilevers for VOC sensors. Sensors and Actuators, A: Physical 202, 233-239 (2013). *PC Cantilever.*
41. Urwyler, P. et al. Surface patterned polymer micro-cantilever arrays for sensing. Sensors and Actuators, A: Physical 172, 2-8 (2011). *PVDF Cantilever.*

42. Urwyler, P. et al. Surface patterned polymer micro-cantilever arrays for sensing. Sensors and Actuators, A: Physical 172, 2-8 (2011). *PP Cantilever.*
43. Yoon, Y., Chae, I., Thundat, T. & Lee, J. Hydrogel Microelectromechanical System (MEMS) Resonators: Beyond Cost-Effective Sensing Platform. Advanced Materials Technologies 4 (2019). *Membrane.*
44. Thuau, D. et al. Engineering polymer MEMS using combined microfluidic pervaporation and micro-molding. Microsystems and Nanoengineering 4 (2018).
45. Roppolo, I. et al. 3D printable light-responsive polymers. Materials Horizons 4, 396-401 (2017).
46. Stassi, S. et al. Polymeric 3D Printed Functional Microcantilevers for Biosensing Applications. ACS Applied Materials and Interfaces 9, 19193-19201 (2017).
47. Grall, S., Dufour, I., Aubry, V. & Debéda, H. Fabrication and characterisation of piezoelectric screen-printed in plane resonant microcantilevers used as gravimetric sensors. Smart Mater. Struct. 28 (2019).

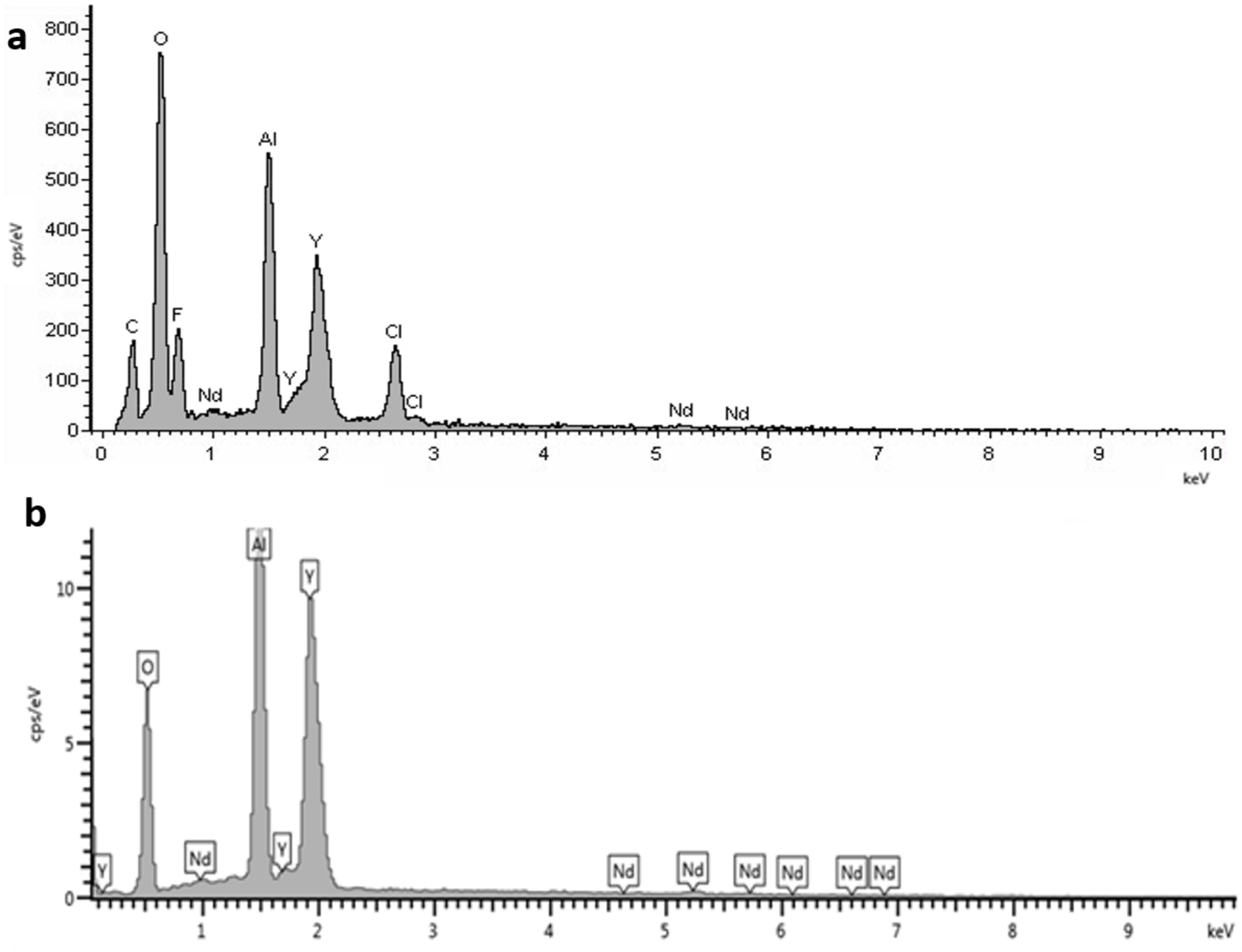

Supplementary Figure S4. Energy Dispersive X-ray (EDX) Analysis of a 3D printed device before thermal curing. The dominant peaks are related to the yttrium, aluminum, oxygen, carbon, and chlorine which come from the precursor used for the formulation of polymerizable ceramic ink. Fluorine peak is related to residuals from the washing step of 3D printed structures with Novec 7100 (composed of methoxy-nonafluorobutane). Lower peaks are related to neodymium (Nd) which is the YAG dopant. **b** EDX of the 3D printed device after the thermal process. The dominant peaks are related to the yttrium, aluminum, and oxygen which are the components of the yttrium aluminum garnet crystals (YAG, $Y_3Al_5O_{12}$) formed during the heating process. Lower peaks related to neodymium (Nd) which is the YAG dopant are still present. The absence of carbon C confirms the complete removal of the organic compound and the conversion into a ceramic structure. Chlorine and fluorine contaminants have been also removed during the heating process.

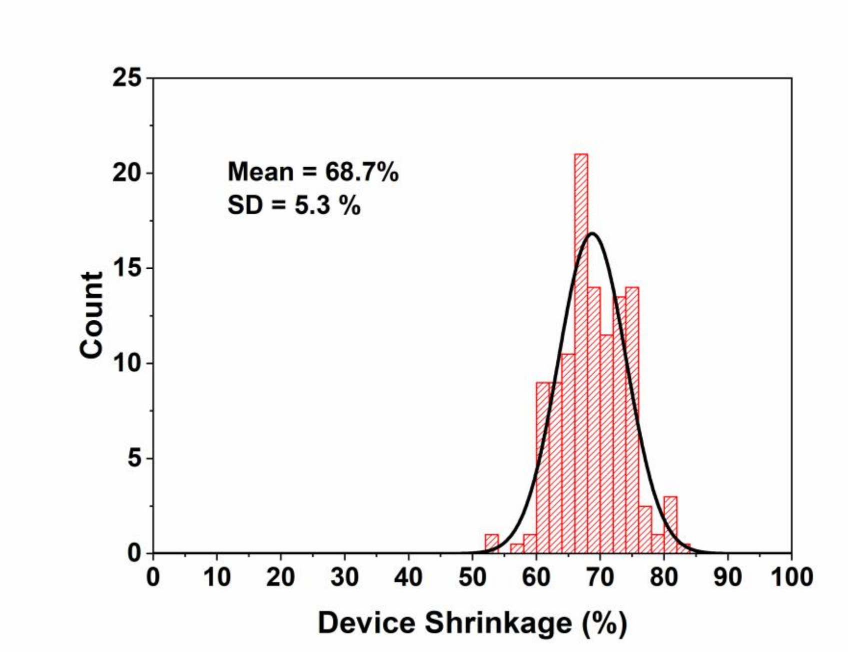

Supplementary Figure S5. Histogram reporting the device dimension shrinkage (ratio between measured dimensions and the theoretical ones used for printing) after thermal curing process measured with SEM analysis. The analysis is computed over more than 200 resonators. Gaussian fit reports a mean value of 68.7 % of device shrinkage with a standard deviation of 5.3%

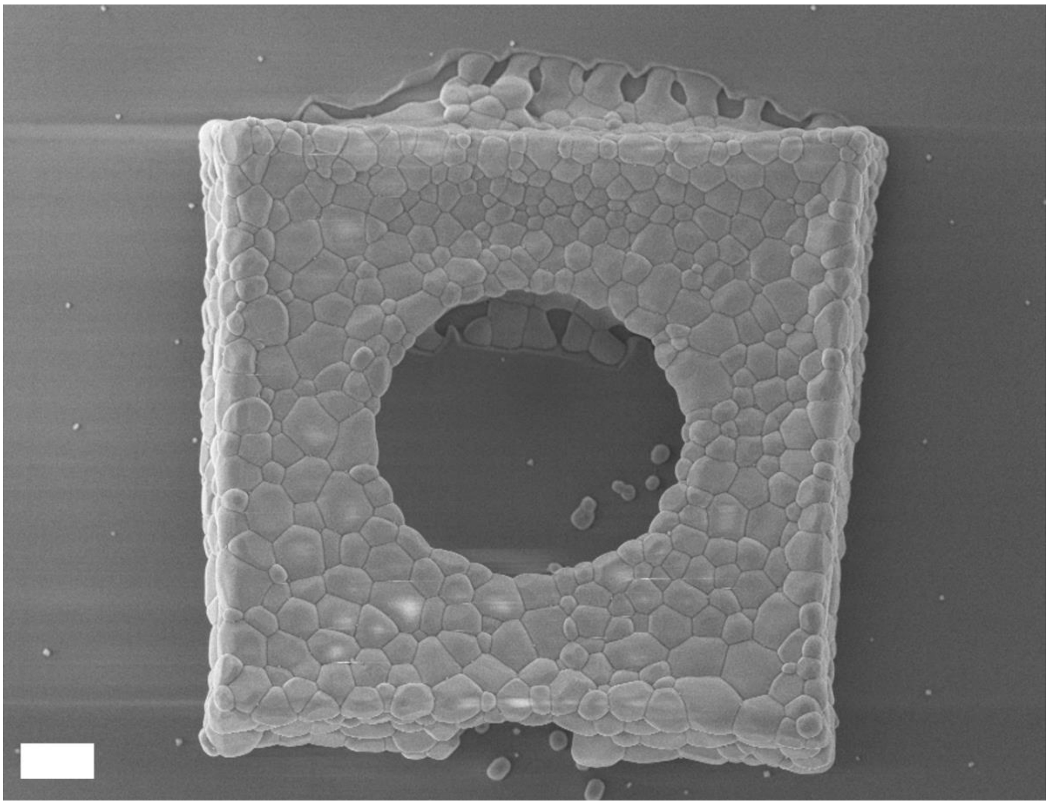

Supplementary Figure S6. SEM image of a membrane device broken by thermal stress induced by annealing process for the densification and the crystallization of the photopolymerized precursor

Supplementary note 2 – Young's modulus evaluation

The elastic properties of the converted printed material (Young's modulus) are evaluated by comparing the value of the experimental resonance frequency of the device (Figure 3b) obtained with a driven measurement with the theoretical prediction from equation 1. This analysis confirms that the printed devices have a Young's modulus in line with the literature value for Nd:YAG of 290 GPa.

2.1 Thermal noise

An additional evaluation of material Young's modulus is performed by extrapolating the effective stiffness (or spring constant) of the devices from the measurement of thermal noise spectra. From the effective stiffness is then possible to obtain the material Young's modulus.

The effective stiffness of the device can be extrapolated using the equipartition theorem from the mean squared displacement of the resonator's thermal motion $\langle {x_{th}}^2 \rangle$ as:

$$\frac{1}{2} k_{eff} \langle {x_{th}}^2 \rangle = \frac{1}{2} k_B T \text{ (1)}$$

$$k_{eff} = \frac{k_B T}{\langle {x_{th}}^2 \rangle} \text{ (2)}$$

where k_B is the Boltzmann constant and T the absolute temperature. $\langle {x_{th}}^2 \rangle$ is experimentally measured integrating the area under the resonance peak of the thermal noise spectrum[1].

Then by comparing the theoretical resonance frequency f_0 of the resonator from the Eulero-Bernoulli beam theory (Equation 1 of the main article):

$$f_0 = A(E/\rho)^{1/2} t/L^2 \quad \text{(3)}$$

with the theoretical resonance frequency from a lumped-element model resonator[2]:

$$f_0 = \frac{1}{2\pi} \sqrt{\frac{k_{eff}}{m_{eff}}} \quad \text{(4)}$$

where m_{eff} is the effective resonator mass (for a cantilever at fundamental resonance mode is *0.25LWTρ),* the dependence of the Young's modulus from the effective stiffness results as:

$$E = \frac{1}{A^2 \pi^2} \frac{L^3}{wt^3} k_{eff} \text{ (5)}$$

The evaluation of Young's modulus from thermal noise spectra has been conducted on less devices than the driven analysis, because the measurement of resonance peak from thermal noise is experimentally complex on small devices with resonance frequencies in the MHz regime. Very small amplitude vibration and elevate contribution of instrumental noise reduce the detection of thermal noise peak and introduce high uncertainty in the Lorentzian peak fitting. We obtained a Young's modulus from thermal noise spectra evaluation of 268 ± 59 GPa (see Supplementary Figure S7) which is in line with the Nd:YAG tabulated material value (290 GPa) and the results of drive measurement analysis (fit in Figure 3b).

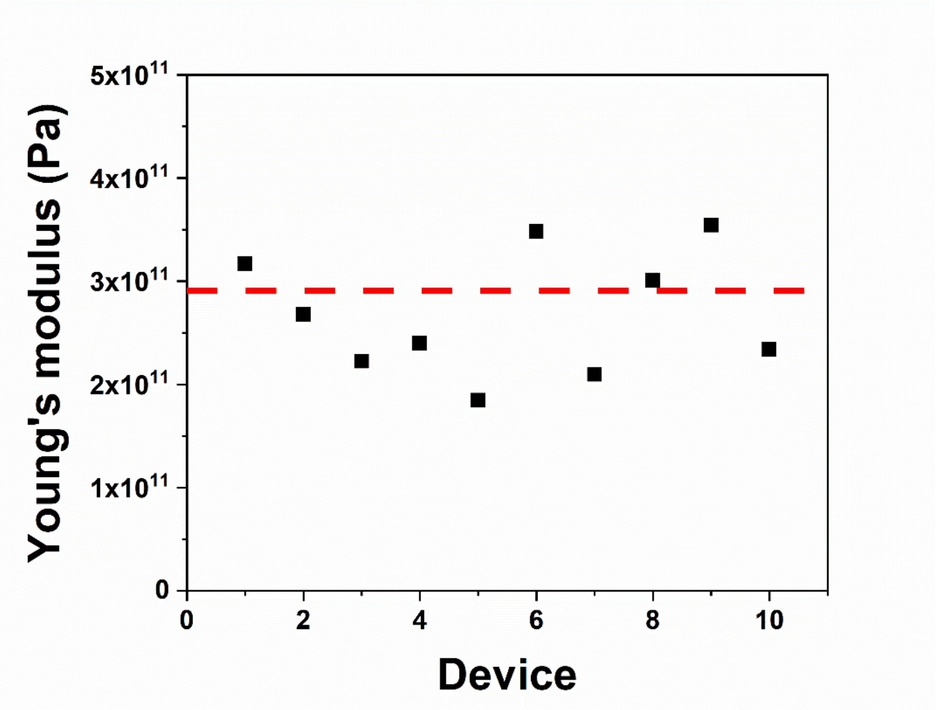

Supplementary Figure S7 Young's modulus for different devices evaluated by thermal noise method.

2.2 Nanoindentation

The Young's modulus was also determined by carrying out a static mechanical analysis by means of AFM nanoindentation measurements. A diamond nanoindenting probe (Bruker DNISP-HS) was employed for acquiring the force curves by using an *Innova* AFM from Bruker. The tip consists of the corner of a diamond cube, with a curvature radius of the tip apex equal to 40 nm. The cantilever is made of stainless steel and the spring constant, calibrated at the factory, is equal to $k = 353\ \mathrm{N/m}$. The sensitivity of the cantilever (nanometers of cantilever deflection as a function of photodetector voltage variation) is determined before each measurement session by using a sapphire sample. In this set of measurements, the determined sensitivity was $s = 42.6\ \mathrm{nm/V}$. A fused silica sample was used as a reference test for the accuracy of the modulus determination. The force curves are acquired by using the Bruker Nanodrive software (the same used for the topography mapping) and analyzed with Matlab. The deflection, D vs piezo displacement, z curves are converted into force vs separation, δ curves, where the separation is the amount of penetration of the tip into the sample. The deflection is measured in Volts and converted into force as $F = D \cdot s \cdot k$ while δ is obtained as $\delta = z - (D \cdot s)$. Given that the measured specimens are stiff and there is negligible adhesion between the tip and the sample, the Hertz model[3,4] was adopted for fitting the retract part of the curves and determining the Young's modulus. The Hertz formula that describes the force as a function of the tip penetration into the sample is:

$$F = \frac{4}{3} E_r \sqrt{R} \delta^{\frac{3}{2}} \quad (6)$$

where R is the tip radius and E_r is the reduced Young's modulus, given by:

$$\frac{1}{E_r} = \frac{1 - \nu_t^2}{E_t} + \frac{1 - \nu_s^2}{E_s} \quad (7)$$

where E is the Young's modulus, ν is the Poisson ratio and the subscripts t and s refer to the tip and sample, respectively. For the tip, we used $E_t = 1140\ \mathrm{GPa}$ and $\nu_t = 0.2$. For the analysis, we used the linearized model and fitted the curves as

$$F^{\frac{2}{3}} = \left(\frac{4}{3} E_r \sqrt{R}\right)^{\frac{2}{3}} \delta \quad (8)$$

so that the fitting function is a line, and the reduced modulus is obtained from the slope, avoiding the need to precisely determining the contact point. We always fitted the retract curves in the 25%-90% range to take as much as possible into account only the elastic deformations of the sample and

to avoid possible mechanical adjustments between the tip and the sample during the approach or during the final part of the retract curve.

Supplementary Figure S8a reports a topography map obtained on a membrane. Since the nanoindenting tip is not meant for imaging, the topography contains artefacts due to the convolution between the sample and the tip shape. Indeed, the height of the tip is $50\ \mu m$, the cantilever is slightly inclined with respect to the surface of the sample and the scan was performed by acquiring 256 horizontal lines starting from the bottom. Therefore, it can be noticed that on the sides but especially on the top edge of the membrane the apparent slowly decreasing height of the sample is due to the fact that the lateral face of the pyramidal diamond tip is touching the edge of the sample. The map was merely used for helping to select the points on which to acquire the force curves, which were taken close to the edges of the sample.

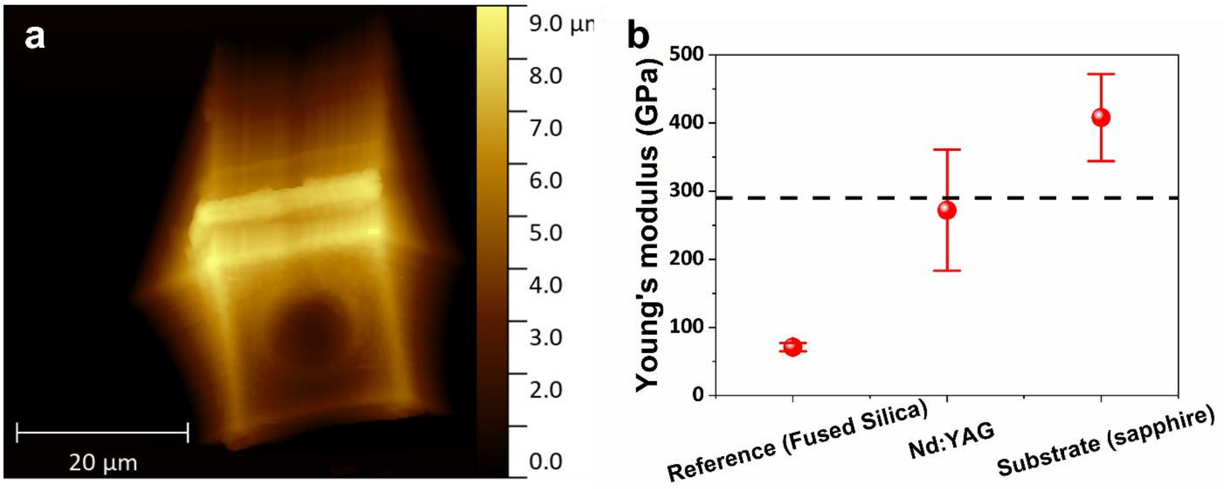

Supplementary Figure S8. **a** Topography map of a membrane device obtained with the diamond nanoindentation probe. Several force curves were obtained on the edge of the membrane and analyzed with the Hertz model. The probe is not meant for imaging: the apparent slow decrease in height close to the top and laterals sides is due to the convolution of the probe shape with the sample. **b** Young's modulus values obtained by analyzing with the Hertz model the force curves obtained from the device shown in panel **a**. Young's moduli for the substrate and for a fused silica reference sample are shown as well.

Supplementary Figure S8b reports the Young's modulus value obtained by averaging over 30 different values obtained from as many force curves on the YAG membrane. The result was $E = 272 \pm 89$ GPa, by assuming a Poisson ratio equal to $\nu = 0.275$. E is therefore in good agreement with that reported in literature for Nd:YAG[5,6]. The rather large uncertainty, calculated as the standard deviation, is due to the fact that the measurement is local and can be particularly sensitive to the presence of crystalline grains and grain boundaries (see Figure S6). Some force curves were also acquired on the sapphire substrate close to the membrane. Also in this case the obtained value, $E = 408 \pm 64$ GPa (with $\nu = 0.27$), is in the correct range for this material[7], while the significant uncertainty is mainly given to the fact that, the higher the Young's modulus, the more sensitive is the obtained value on the slope of the force curves. The Young's modulus obtained from 30 force curves acquired on a reference fused silica sample is also reported in the same figure, to check the accuracy of the measurements. The obtained value in this case was $E = 71 \pm 6$ GPa ($\nu = 0.16$), in agreement with the expected modulus for this material[8].

Supplementary note 3 – Quality factor contribution

For the evaluation of the theoretical quality factor of our resonators we take in account all the possible dissipation mechanism which are involved in the damping of the mechanical vibration of our structures. The total contribution will be extrapolated by the sum of all the damping mechanism, by considering their contribution as the inverse of *Q*. The dissipation mechanism in our devices are gas damping (Q_{gas}), clamping loss (Q_{clamp}), internal friction (Q_{frict}), surface loss (Q_{surf}) and thermoelastic damping (Q_{TED})[2].

$$\frac{1}{Q} = \frac{1}{Q_{gas}} + \frac{1}{Q_{clamp}} + \frac{1}{Q_{frict}} + \frac{1}{Q_{surf}} + \frac{1}{Q_{TED}} \quad (9)$$

Gas damping (Q_{gas}) in high vacuum regime is the dissipation mechanism due by the interaction of air particles with the resonator device. Two separate contribution are present, drag-force damping ($Q_{gas\text{-}df}$), caused by collision with air molecules, and squeeze-film damping ($Q_{gas\text{-}sf}$), due to the squeeze of air film between the structure and the lower surface. Both contributions are linearly dependent with the device thickness *t* as:

$$Q_{gas-df} = \frac{\rho t \omega}{4p} \sqrt{\frac{\pi R T}{2M}} \quad (10)$$

$$Q_{gas-sf} = (2\pi)^{\frac{3}{2}} \frac{\rho t \omega d_0}{4pL} \sqrt{\frac{RT}{M}} \quad (11)$$

where ρ, ω, L are the resonator density, eigenfrequency and length, respectively, p the air pressure, T the temperature, d_0 the gap between surface and the device, M the gas molar mass and R the universal gas constant. Both damping mechanisms give a negligible *Q* contribution since their values are always above 10^{10} for our resonators[9].

Clamping loss (Q_{clamp}) are related to the energy loss at the clamping of the resonating device with the bulk. Since we have a sufficiently thick supporting structures, the damping contribution can be evaluated as:

$$Q_{clamp} = \left(0.31\frac{w}{L}\left(\frac{t}{L}\right)^4\right)^{-1} \quad (12)$$

where w is the device width. Even if the clamping loss follow a t^{-4} dependence, the contribution in our device is always far below the other damping mechanism (Q_{clamp}>10^5)[10].

Internal friction (Q_{frict}) are due to the atoms motions during the device vibrations and thus to material viscoelasticity[11]. Friction losses are computed directly by the ratio between the real (E) and imaginary (E') part of Young's modulus which is the definition of the inverse of the loss tangent (tan δ):

$$Q_{frict} = \frac{E}{E'} = (\tan\delta)^{-1} \quad (13)$$

Polymeric materials have loss tangent in the range 10^{-1}-10^{-2} and then friction loss results as the main dissipation mechanism in standard 3D printed device and in our NEMS device before thermal curing. Ceramic materials like aluminum oxides and thus garnet (YAG) exhibits loss factor of around 10^{-5} which give a Q contribution of 10^5. Therefore, after thermal curing, the contribute to overall damping given by internal friction in our resonators becomes very small.

Surface loss (Q_{surf}) and **thermoelastic damping** (Q_{TED}) mechanisms are described in detail in the manuscript.

All the dissipation mechanism contributes in our devices are reported in Supplementary Figure S9 evidencing as Q_{surf} and Q_{TED} dominates over the other loss factors.

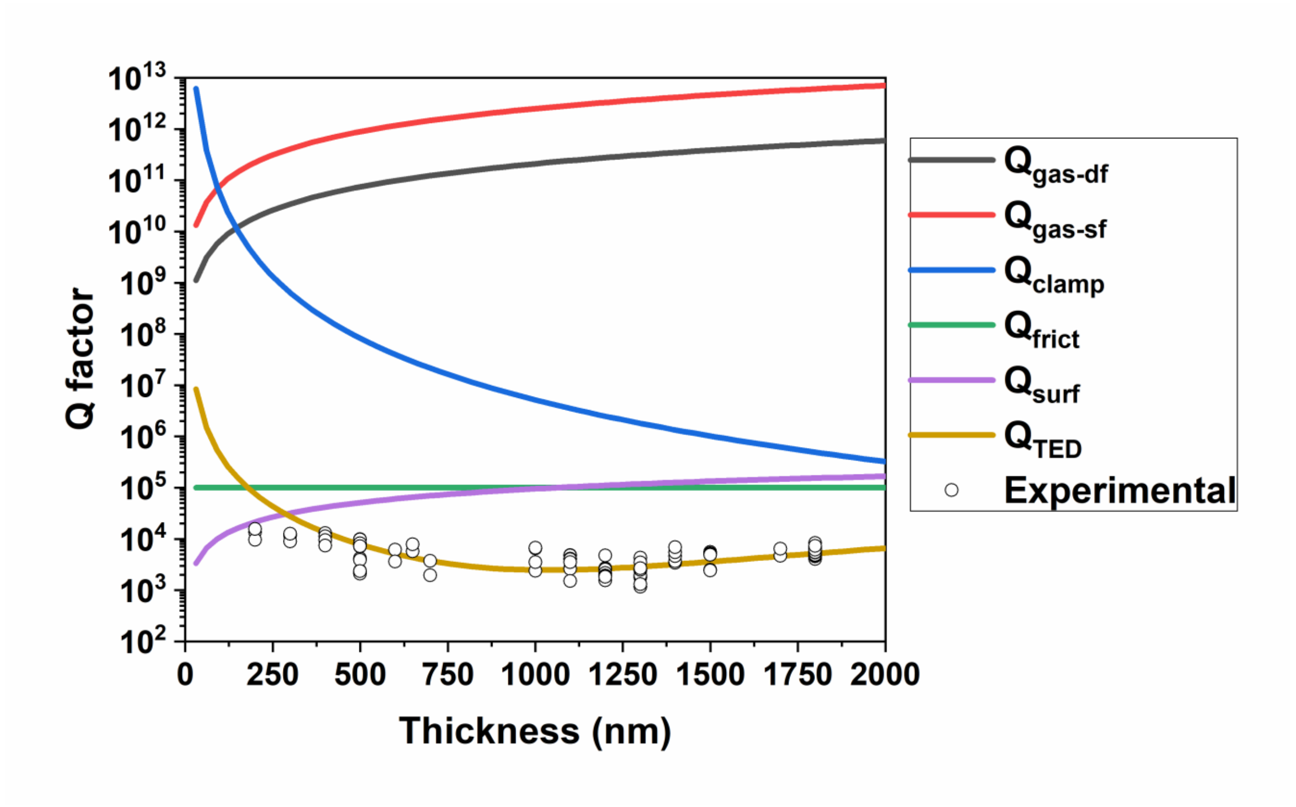

Supplementary Figure S9. Thick lines represent the quality factor contribution by the different dissipation mechanism as function of device thickness in our nanomechanical resonators. Circle point represents the experimentally measured Q factor of 3D printed nanomechanical resonators.

Supplementary note 4 – Frequency stability review

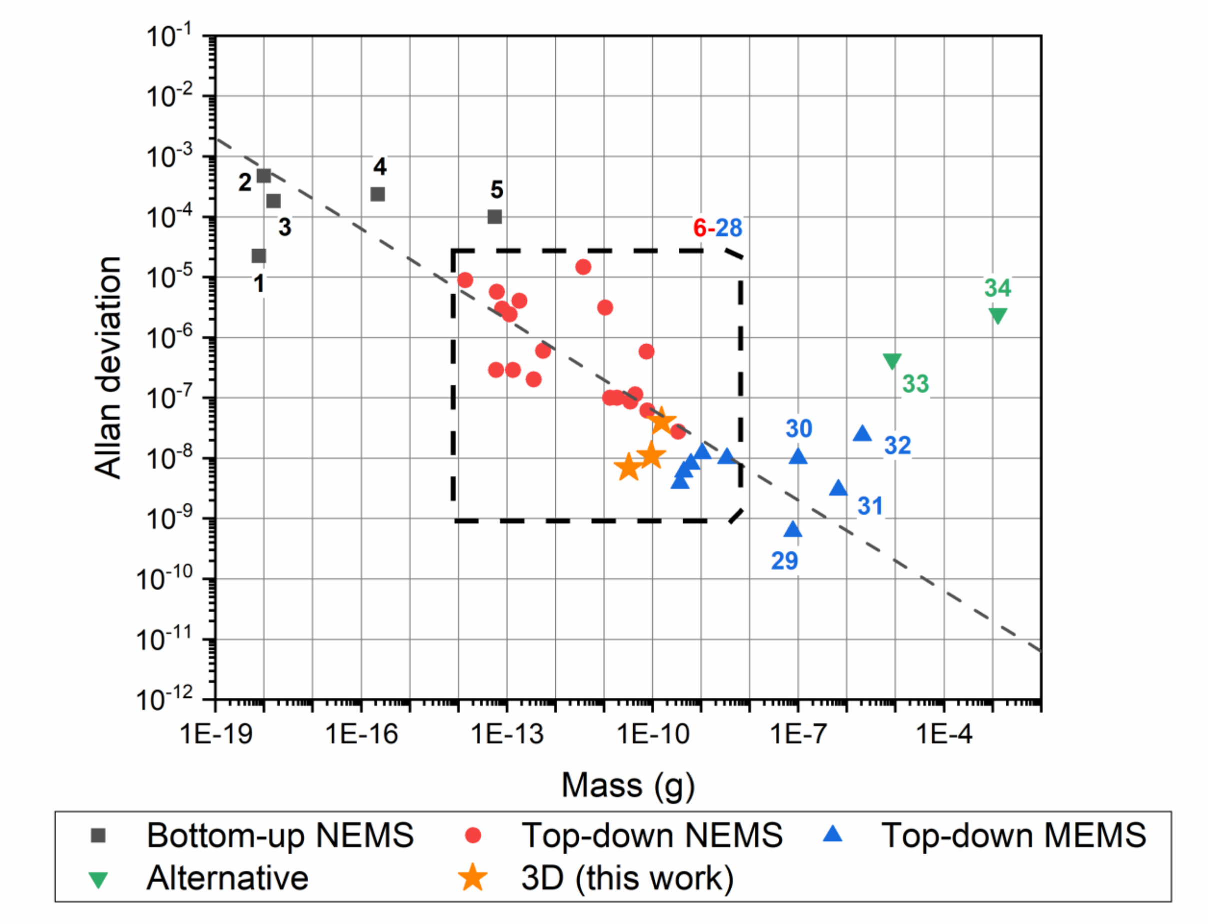

Supplementary Figure S10. Plot of Figure 4e of the manuscript with the mapping of the reference work from which frequency stability (minimum of Allan deviation) data are extracted. The reference articles are reported below. The dashed rectangle represents the zoomed area of Supplementary Figure S11.

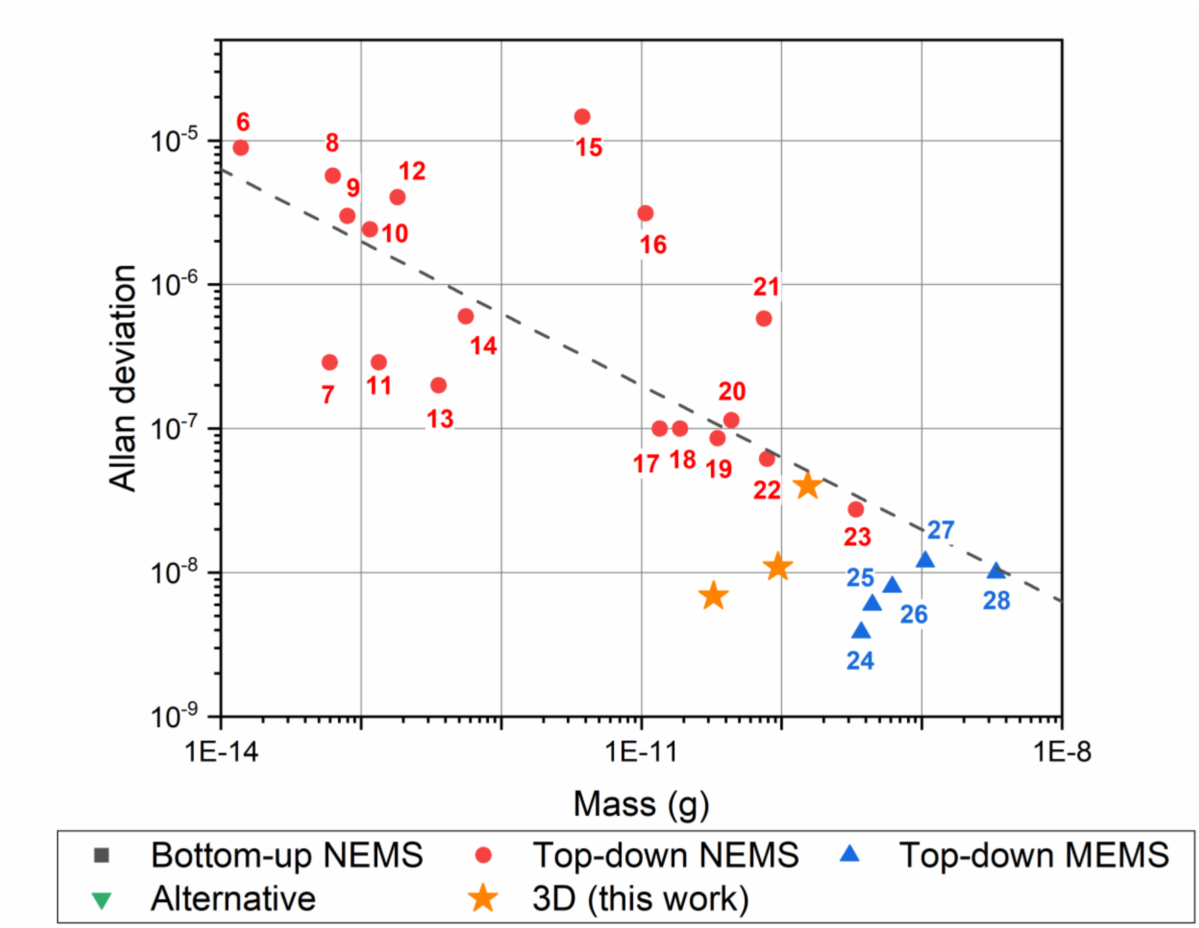

Supplementary Figure S11. Zoom of Supplementary Figure S10 containing reference points from 6 to 28.

Figure 4e of the manuscript reports the frequency stability values of several mechanical resonators extrapolated from literature works and compare to the performance of our devices. The figure is an integrated version of the analysis presented by Sansa *et al.* with our devices (stars) and more recent literature works. The devices are divided in the same 4 different categories related to fabrication technology and resonator dimensions used for the quality factor analysis. The literature works between quality factor and frequency stability do not totally coincide since many articles report only the *Q* values, especially the articles related to alternative approaches. Below is reported the reference list of the numbered points of Supplementary Figure S10 and S11 (some works presented more than one type of resonator).

BOTTOM-UP NEMS

1. Chaste, J. et al. A nanomechanical mass sensor with yoctogram resolution. Nature Nanotechnology 7, 301-304 (2012).
2. Chiu, H. Y., Hung, P., Postma, H. W. C. & Bockrath, M. Atomic-scale mass sensing using carbon nanotube resonators. Nano Lett. 8, 4342-4346 (2008).
3. Jensen, K., Kim, K. & Zettl, A. An atomic-resolution nanomechanical mass sensor. Nature Nanotechnology 3, 533-537 (2008).
4. Chen, C. et al. Performance of monolayer graphene nanomechanical resonators with electrical readout. Nature Nanotechnology 4, 861-867 (2009).
5. Stassi, S. et al. Nanomechanical DNA resonators for sensing and structural analysis of DNA-ligand complexes. Nat. Commun. 10, 1690 (2019).

TOP-DOWN NEMS

6. Bartsch, S. T., Rusu, A. & Ionescu, A. M. Phase-locked loop based on nanoelectromechanical resonant-body field effect transistor. Appl. Phys. Lett. 101 (2012).
7. Sansa, M., Fernández-Regúlez, M., Llobet, J., San Paulo, Á. & Pérez-Murano, F. High-sensitivity linear piezoresistive transduction for nanomechanical beam resonators. Nat. Commun. 5 (2014). *Bridge bottom-up.*
8. Sage, E. *et al.* Single-particle mass spectrometry with arrays of frequency-addressed nanomechanical resonators. *Nat. Commun.* **9** (2018).
9. Ramos, D. et al. Optomechanics with silicon nanowires by harnessing confined electromagnetic modes. Nano Lett. 12, 932-937 (2012).
10. Feng, X. L., White, C. J., Hajimiri, A. & Roukes, M. L. A self-sustaining ultrahigh-frequency nanoelectromechanical oscillator. Nature Nanotechnology 3, 342-346 (2008).
11. Sansa, M., Fernández-Regúlez, M., Llobet, J., San Paulo, Á. & Pérez-Murano, F. High-sensitivity linear piezoresistive transduction for nanomechanical beam resonators. Nat. Commun. 5 (2014). *Bridge top-down.*
12. Yang, Y. T., Callegari, C., Feng, X. L., Ekinci, K. L. & Roukes, M. L. Zeptogram-scale nanomechanical mass sensing. Nano Lett. 6, 583-586 (2006).
13. Sansa, M. et al. Frequency fluctuations in silicon nanoresonators. Nature Nanotechnology 11, 552-558 (2016).
14. Mile, E. et al. In-plane nanoelectromechanical resonators based on silicon nanowire piezoresistive detection. Nanotechnology 21 (2010).

15. Gray, J. M., Bertness, K. A., Sanford, N. A. & Rogers, C. T. Low-frequency noise in gallium nitride nanowire mechanical resonators. Appl. Phys. Lett. 101 (2012).

16. Villanueva, L. G. et al. A nanoscale parametric feedback oscillator. Nano Lett. 11, 5054-5059 (2011).

17. Verd, J. et al. Monolithic CMOS MEMS oscillator circuit for sensing in the attogram range. IEEE Electron Device Letters 29, 146-148 (2008).

18. Gavartin, E., Verlot, P. & Kippenberg, T. J. Stabilization of a linear nanomechanical oscillator to its thermodynamic limit. *Nat. Commun.* **4** (2013).

19. Gagino, M. et al. Suspended Nanochannel Resonator Arrays with Piezoresistive Sensors for High-Throughput Weighing of Nanoparticles in Solution. ACS Sensors 5, 1230-1238 (2020).

20. Verd, J. *et al.* Design, fabrication, and characterization of a submicroelectromechanical resonator with monolithically integrated CMOS readout circuit. *Journal of Microelectromechanical Systems* **14**, 508-519 (2005).

21. Verd, J. *et al.* Design, fabrication, and characterization of a submicroelectromechanical resonator with monolithically integrated CMOS readout circuit. *Journal of Microelectromechanical Systems* **14**, 508-519 (2005).

22. Fong, K. Y., Pernice, W. H. P. & Tang, H. X. Frequency and phase noise of ultrahigh Q silicon nitride nanomechanical resonators. *Physical Review B - Condensed Matter and Materials Physics* **85** (2012).

23. Park, K. K. *et al.* Capacitive micromachined ultrasonic transducer (CMUT) as a chemical sensor for DMMP detection. *Sensors and Actuators, B: Chemical* **160**, 1120-1127 (2011).

TOP-DOWN MEMS

24. Olcum, S. et al. Weighing nanoparticles in solution at the attogram scale. Proceedings of the National Academy of Sciences of the United States of America 111, 1310-1315 (2014). *Cantilever 22.5 µm long.*

25. Olcum, S. et al. Weighing nanoparticles in solution at the attogram scale. Proceedings of the National Academy of Sciences of the United States of America 111, 1310-1315 (2014). *Cantilever 27 µm long.*

26. Olcum, S. et al. Weighing nanoparticles in solution at the attogram scale. Proceedings of the National Academy of Sciences of the United States of America 111, 1310-1315 (2014). *Cantilever 37.5 µm long.*

27. Olcum, S. et al. Weighing nanoparticles in solution at the attogram scale. Proceedings of the National Academy of Sciences of the United States of America 111, 1310-1315 (2014). *Cantilever 50 µm long.*

28. Ramos, D., Malvar, O., Davis, Z. J., Tamayo, J. & Calleja, M. Nanomechanical Plasmon Spectroscopy of Single Gold Nanoparticles. Nano Lett. 18, 7165-7170 (2018).

29. Ivaldi, P. *et al.* 50 nm thick AlN film-based piezoelectric cantilevers for gravimetric detection. *Journal of Micromechanics and Microengineering* **21** (2011).

30. Burg, T. P. et al. Weighing of biomolecules, single cells and single nanoparticles in fluid. Nature 446, 1066-1069 (2007).
31. Stassi, S. et al. Large-scale parallelization of nanomechanical mass spectrometry with weakly-coupled resonators. Nat. Commun. 10 (2019).
32. Calmo, R. et al. Monolithic glass suspended microchannel resonators for enhanced mass sensing of liquids. Sensors and Actuators, B: Chemical, 298-303 (2019).

ALTERNATIVE APPROACHES

33. Yoon, Y., Chae, I., Thundat, T. & Lee, J. Hydrogel Microelectromechanical System (MEMS) Resonators: Beyond Cost-Effective Sensing Platform. Advanced Materials Technologies 4 (2019).
34. Grall, S., Dufour, I., Aubry, V. & Debéda, H. Fabrication and characterisation of piezoelectric screen-printed in plane resonant microcantilevers used as gravimetric sensors. Smart Mater. Struct. 28 (2019).

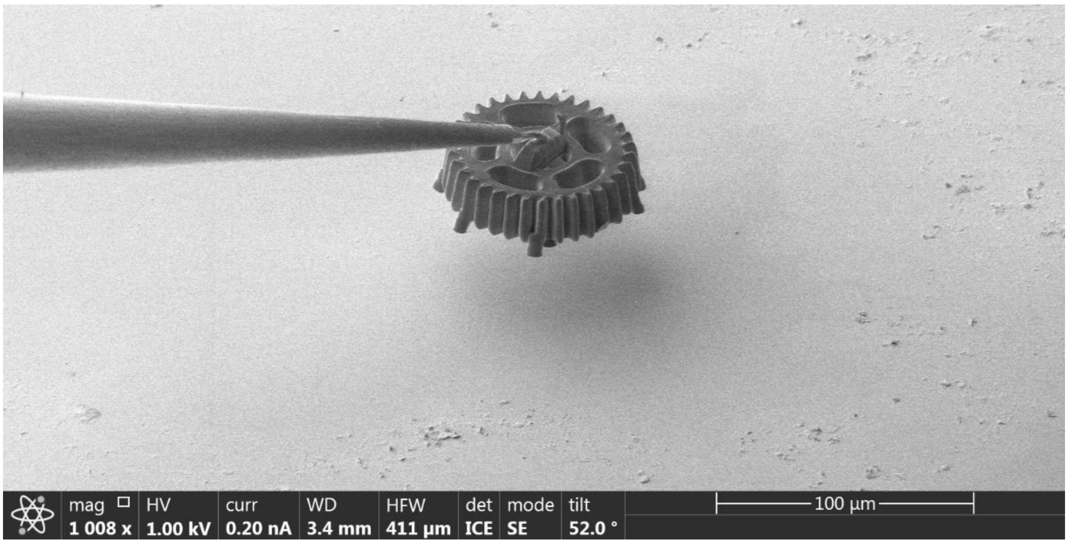

Supplementary Figure S12. SEM image of lifting a printed YAG structure with a pick-up setup available within a Focused Ion Beam (FIB) instrument.

References

1 Ohler, B. Cantilever spring constant calibration using laser Doppler vibrometry. *Review of Scientific Instruments* **78** (2007).

2 Schmid, S., Villanueva, L. G. & Roukes, M. L. *Fundamentals of nanomechanical resonators*. (Springer International Publishing, 2016).

3 Hertz, H. Ueber die Berührung fester elastischer Körper. **1882**, 156-171 (1882).

4 Butt, H.-J., Cappella, B. & Kappl, M. Force measurements with the atomic force microscope: Technique, interpretation and applications. *Surface Science Reports* **59**, 1-152 (2005).

5 Mezeix, L. & Green, D. J. Comparison of the Mechanical Properties of Single Crystal and Polycrystalline Yttrium Aluminum Garnet. *International Journal of Applied Ceramic Technology* **3**, 166-176 (2006).

6 Li, J. *et al.* Fabrication, microstructure and properties of highly transparent Nd:YAG laser ceramics. *Optical Materials* **31**, 6-17 (2008).

7 Vodenitcharova, T. *et al.* The effect of anisotropy on the deformation and fracture of sapphire wafers subjected to thermal shocks. *Journal of Materials Processing Technology* **194**, 52-62 (2007).

8 Zheng, L., Schmid, A. W. & Lambropoulos, J. C. Surface effects on Young's modulus and hardness of fused silica by nanoindentation study. *Journal of Materials Science* **42**, 191-198 (2007).

9 Bao, M., Yang, H., Yin, H. & Sun, Y. Energy transfer model for squeeze-film air damping in low vacuum. *Journal of Micromechanics and Microengineering* **12**, 341-346 (2002).

10 Photiadis, D. M. & Judge, J. A. Attachment losses of high Q oscillators. *Applied Physics Letters* **85**, 482-484 (2004).

11 Tsaturyan, Y., Barg, A., Polzik, E. S. & Schliesser, A. Ultracoherent nanomechanical resonators via soft clamping and dissipation dilution. *Nature Nanotechnology* **12**, 776-783 (2017).
